# Prenatal exposure to antibiotics and risk of neurodevelopmental disorders in offspring: A systematic review and meta-analysis

**DOI:** 10.3389/fneur.2022.1045865

**Published:** 2022-11-25

**Authors:** Qiuji Tao, Yajun Shen, Yang Li, Huan Luo, Meng Yuan, Jing Gan

**Affiliations:** ^1^Department of Pediatrics of Neurology Nursing, West China School of Nursing, West China Second University Hospital, Sichuan University, Chengdu, Sichuan, China; ^2^Key Laboratory of Birth Defects and Related Diseases of Women and Children (Sichuan University), Ministry of Education, Chengdu, Sichuan, China; ^3^Department of Pediatrics, West China Second University Hospital, Sichuan University, Chengdu, Sichuan, China; ^4^Key Laboratory of Obstetric and Gynecologic and Pediatric Diseases and Birth Defects of Ministry of Education, Sichuan University, Chengdu, Sichuan, China; ^5^Key Laboratory of Development and Maternal and Child Diseases of Sichuan Province, Chengdu, Sichuan, China

**Keywords:** prenatal antibiotic exposure, neurodevelopment disorder, autism, attention-deficit/hyperactivity deficit, cerebral palsy, epilepsy

## Abstract

**Background and purpose:**

A growing body of research suggests that inflammation and maternal infections may lead to an increased risk of neurodevelopmental problems such as attention-deficit/hyperactivity disorder (ADHD), autism spectrum disorder (ASD), cerebral palsy (CP), and epilepsy in offspring. The aim of this study was to observe the connection between prenatal antibiotic exposure and the risk of these neurodevelopmental disorders in offspring.

**Patients and methods:**

A comprehensive search was conducted in the Cochrane Central Register of Controlled Trials (CENTRAL), PubMed, Google Scholar, and Scopus databases for observational studies that looked into the link between prenatal exposure to antibiotics and the risk of neurodevelopmental problems in offspring, published from 1 January 1950 to 31 January 2022. The Newcastle–Ottawa Scale (NOS) was used to assess the quality of the included studies. Data were analyzed using the STATA version 12 software, and an odds ratio (OR) with a 95% confidence interval (CI) was reported.

**Results:**

A total of 15 studies were included in the meta-analysis. Prenatal antibiotic exposure was associated with the increased risk of ADHD (OR = 1.14; 95% CI = 1.13 to 1.15; *I*^2^ = 0%) and epilepsy (OR = 1.34; 95% CI = 1.02 to 1.66; *I*^2^ = 96.8%). The link between prenatal antibiotic exposure and the risk of ASD [OR = 1.09; 95 % CI = 0.88 to 1.31; *I*^2^ = 78.9%] and CP [OR = 0.99; 95% CI = 0.56 to 1.43; *I*^2^ = 91%] was found to be non-significant. In all of the included prospective cohort studies, subgroup analysis suggested a significant association between prenatal antibiotic exposure and the incidence of ASD [OR = 1.17; 95% CI = 1.03 to 1.31; *I*^2^ = 48.1%] and CP [OR = 1.18; 95% CI = 1.02 to 1.34; *I*^2^ = 0%].

**Conclusion:**

Prenatal antibiotic exposure during pregnancy is linked to a higher incidence of ADHD and epilepsy in the offspring. Further prospective studies that compare prenatal antibiotic use and are adjusted for various confounders are needed to further assess the association of prenatal antibiotic exposure and neurological disorders in offspring.

**Systematic review registration:**

https://www.crd.york.ac.uk/prospero/, identifier: CRD42022306248.

## Introduction

A higher risk of neurological disorders has been related to severe viral and bacterial infections, as well as infections caused by parasites, particularly in hospitalized patients (1–3). Continuous complex neurodevelopmental processes such as axonal and dendritic growth, neurogenesis, synaptogenesis, and refinement of these synaptic connections make the prenatal period and infancy particularly vulnerable to exposure (4). Antibiotics, which account for 80% of all drugs provided to pregnant women, are commonly used to treat infections during pregnancy (5). It is projected that 19–49% of pregnant women are given antimicrobials during their pregnancy and that this number will continue to rise. Antibiotic usage during pregnancy has been linked to immune system changes, childhood asthma, changes in gut microbiota, obesity, and functional deficits in the offspring (6–11).

Numerous studies demonstrated that maternal infections and inflammation influence the development of the fetal brain, raising the chance of mental disorders, with a focus on neurodevelopmental diseases (1, 12–14). While some studies have found that the use of antimicrobials during pregnancy is associated with the increased risk of neurodevelopmental disorders including cerebral palsy (CP), attention-deficit/hyperactivity deficit (ADHD), autism spectrum disorder (ASD), and epilepsy, other studies did not find such association (11, 15–20).

Antibiotic exposure during pregnancy has been linked to the likelihood of ASD (21) and ADHD (22) in two recent meta-analyses. These reviews, however, reported data from a small number of studies and could not cover all key neurodevelopmental disorders, such as cerebral palsy and epilepsy. The goal of this systematic review and meta-analysis is to look at the association between prenatal antibiotic exposure and the risk of these neurodevelopmental disorders in offspring.

## Materials and methods

### Study design

This study involves a systematic review and meta-analysis for critical assessment and evaluation of all published reports that evaluate the association of prenatal antibiotic exposure with the risk of neurodevelopmental problems in offspring.

### Ethical clearance

No ethical approval is needed for this systematic review and meta-analysis, as it was based on already published data.

### Protocol and registration

We used the Preferred Reporting Items for Systematic Reviews and Meta-Analyses (PRISMA) statement (23) to conduct the study, with a PROSPERO number (CRD42022306248).

### Eligibility criteria

#### Inclusion criteria

(a) Observational studies investigating the effect of prenatal antibiotic exposure and the risk of neurodevelopmental disorders in offspring. (b) Children diagnosed with any one of the neurodevelopmental disorders including ASD, ADHD, CP, and epilepsy based on prenatal antibiotic exposure. (c) The Control group defined as Offspring with no diagnosis of any neurodevelopmental or other psychiatric or genetic disorders.

#### Exclusion criteria

(a) Duplicate studies, systematic reviews, editorials, case series, case reports, conference abstracts, and preprints. (b) Studies that do not describe required outcomes. (c) Unavailability of full texts. (d) Animal studies. (e) Gray literature (reports, conference proceedings, and theses) and ongoing studies.

### Search strategy

This systematic review was performed following the guidelines of the PRISMA (23) and Cochrane (24). An electronic search in Cochrane Central Register of Controlled Trials (CENTRAL), PubMed, Google Scholar, and Scopus databases was performed for articles published from 1 January 1950 to 31 January 2022, with no language restrictions. The following key terms were used: “antibiotic” OR “antimedicine” OR “antibacterial” AND “Neurodevelopmental disorders” OR “ADHD” OR “Attention- deficit hyperactivity” OR “hyperactivity” OR “inattention” AND “Cerebral Palsy” AND “Autism” AND “Seizure disorders” OR Epilepsy. Reference lists of the retrieved papers, prior meta-analyses, and review articles were manually searched for additional relevant studies.

### Data extraction

Data were collected independently by two reviewers and included author name, country, study period, publication year, study design, diagnostic criteria, exposure assessment, sample size, age at the time of antibiotic exposure, outcomes, and crude or adjusted estimates for disease occurrence.

### Risk of bias assessment

Two authors independently assessed the risk of bias using the Newcastle Ottawa Scale (NOS) (25). This scale is used to assess the quality of non-randomized studies for systematic review and meta-analysis and has three important components: group comparability, study group selection, and determination of the target outcome or exposure. Each study may receive a maximum of eight stars (high quality) to a minimum of 0 stars (poor quality). Any disagreements among the authors on the quality scores were resolved by discussion.

### Statistical analysis

Statistical analysis was done using the STATA version 12 software. For pooled estimates, the odds ratio (OR) with a 95% confidence interval (CI) was generated. The *I*^2^ statistic was used to calculate heterogeneity (26). This test determines how much of the difference in the study results is due to heterogeneity rather than sampling error. *I*^2^ of <50% is seen as inconsequential, and *I*^2^ of over 50% indicates moderate to significant heterogeneity. Publication bias was detected by funnel plot. By eliminating one study at a time, we performed sensitivity analyses to see how a single study affected the total risk estimates. *P* < 0.05 indicated statistical significance.

## Results

### Literature selection

**Figure 1:**
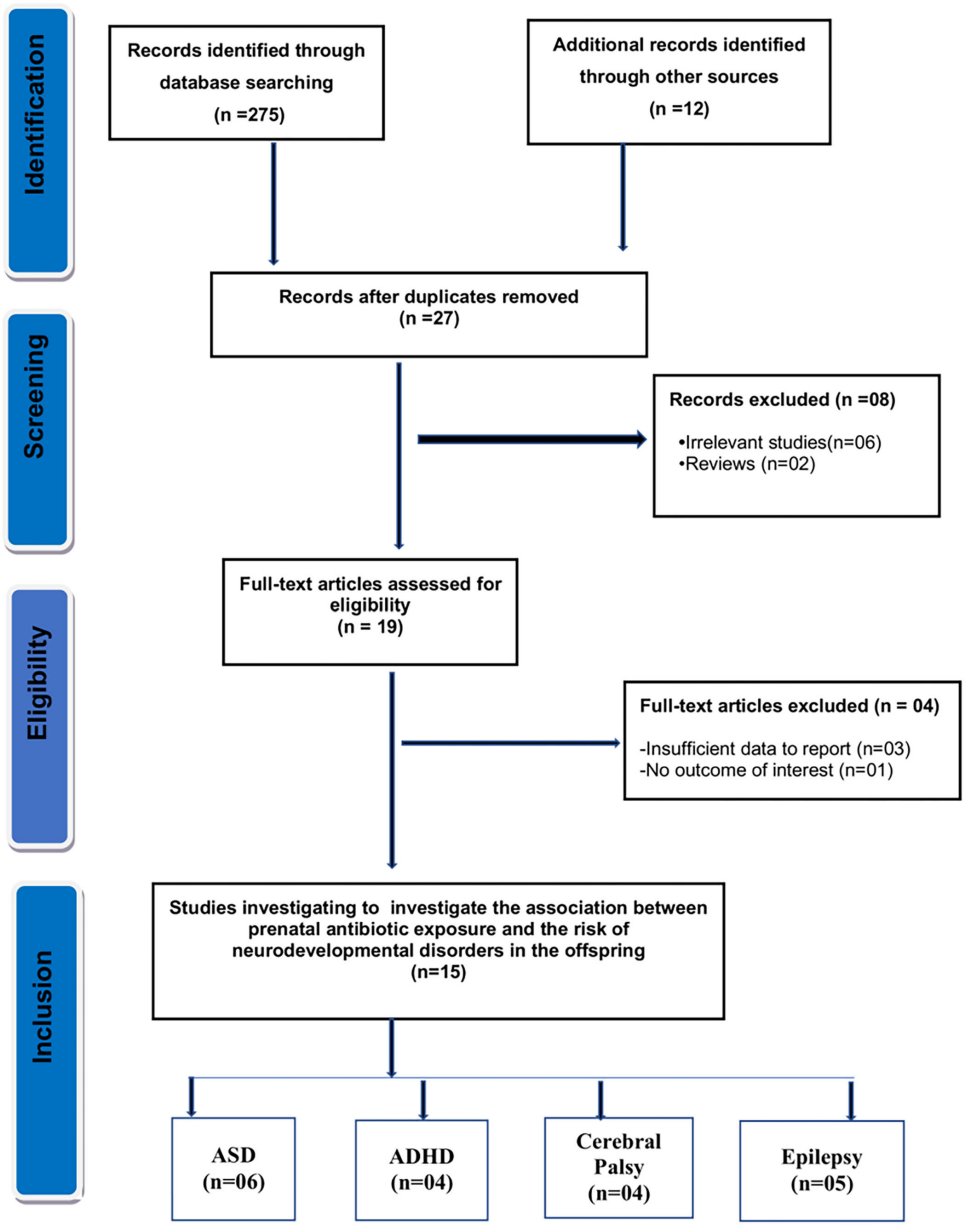
Flow diagram for the selection of studies and specific reasons for exclusion from the present meta-analysis.

As seen in Figure 1, the initial search yielded 287 results. Of them, 27 records were reviewed after duplicate removal, and 19 articles remained after the title and abstract screening. Finally, after evaluating full texts, four articles were removed due to insufficient data or overlapping data, and the remaining 15 studies were selected for the present meta-analysis.

### Study characteristics

Fifteen observational studies (11, 15–20, 27–34) including six studies of ASD (11, 16, 19, 20, 29), four studies of ADHD (16, 17, 27, 28), four studies of CP (15, 16, 33, 34), and five studies of epilepsy (16, 30–32) were included in our systematic review. Three studies were retrospective cohort (15, 17, 30), three studies were case-control (11, 19, 34), one study was nested case-control (20), and the remaining eight studies were prospective cohort studies (16, 18, 27–29, 31–33). The publication year ranged between 1998 and 2021. The baseline and clinical characteristics for the included studies are shown in Table 1. Thirteen included studies were performed in the Caucasian population, and two studies were performed in the Asian population.

**Table 1:** Baseline characteristics of included studies in the meta-analysis investigating the association between prenatal antibiotic exposure and the risk of neurodevelopmental disorders in the offspring.

**S. No**.	**Author, year**	**Country**	**Ethnicity**	**Study design**	**Study period**	**Exposure assessment**	**Diagnosis criteria**	**Sample size**	**Age (Years) for assessment of outcome**	**Antibiotics exposed**	**Endpoints**
1.	Lavebratt et al. (27)	Sweden	Caucasian	PCS	1996-2012	Database Records	ICD-10	990,098	2 to 18	Airway and urinary tract antibiotic	ADHD
2.	Hamad et al. (17)	Canada	Caucasian	RCS	1998-2017	Database Records	AAP	187,605	4 to 18	Penicillin, Other beta lactams, Macrolides and related antibiotics	ADHD
3.	Fan et al. (16)	UK	Caucasian	PCS	1990-2016	Database Records	NA	104,605	0 to 14	Macrolide, Penicillin	ADHD/ASD/ CP/ Epilepsy
4.	Leviton et al. (28)	USA	Caucasian	PCS	NA	Patients Records	NA	583	0 to 10	antibiotic variable conveys information about bacteremia	ADHD
5.	Atladottir et al. (29)	Denmark	Caucasian	PCS	1996-2002		DNBC	976	8 to 14	Penicillin, sulfa drug, other antibiotics or medicine against fungus	ASD
6.	Guisso et al. (11)	Lebanon	Asian	CCS	NA	Patients Records	DSM-IV	314	2 to 18	viral/bacterial infection antibiotics	ASD
7.	Isaksson et al. (19)	Sweden	Caucasian	CCS	NA	Database Records and Survey	Medical History	415	4 to 9	viral/bacterial infection antibiotics	ASD
8.	Abelson et al. (19)	Israel	Asian	NCCS	2008-2006	Database Records	Medical ‘history	451	NA	Antimicrobial	ASD
9.	Hamad et al. (18)	Canada	Caucasian	PCS	1998-2016	Database Records	AAP	2965	1 to 18	viral/bacterial infection antibiotics	ASD
10.	Sassonker-Joseph et al. (30)	Israel	Asian	RCS	1998-2012	Database Records	ICD-9	23471	3 to 18	Macrolides and other antibiotics	Epilepsy
11.	Miller et al. (31)	Denmark	Caucasian	PCS	1996-2004	Database Records	ICD-10	68820	0 to 9.9	Pivmecillinam, sulphamethizole	Epilepsy
12.	Miller et al. (32)	Denmark	Caucasian	PCS	1996-2004	Database Records	ICD-10	172879	0 to 5	Penicillins, sulfonamides and trimethoprim, macrolides, lincosamides, streptogramins, other antibacterials	Epilepsy
13.	O'Shea et al. (34)	USA	Caucasian	CCS	1978-1989	Database Records	Medical History and Bayley Scale	240	0 to 1	Viral/bacterial infection antibiotics	CP
14.	Miller et al. (32)	Denmark	Caucasian	PCS	1997-2003	Database Records	ICD-10	131674	5 to 6	Penicillins, sulfonamides and trimethoprim, macrolides, lincosamides, streptogramins, other antibacterials	CP
15.	Meeraus et al. (15)	UK	Caucasian	RCS	1990-2010	Database Records	NA	64623	0 to 3.6	Bacterial infection antibiotics	CP/Epilepsy

PCS, prospective cohort study; RCS, retrospective cohort study; CCS, case-control study; NCCS, nested case-control study; CP, cerebral palsy; NA, not applicable; ADHD, attention-deficit/hyperactivity deficit; ASD, autism spectrum disorder; ICD, international classification of disease; AAP, American academy of pediatrics; DSM-IV, diagnostic and statistical manual of mental disorders.

Four studies reported international disease classification (ICD)-10 criteria, one study used ICD-9, three studies used medical history, and two studies used American academy of pediatrics guidelines as the diagnostic criteria for defining neurodevelopmental disorders. The detailed classes for the antibiotic exposed in all the included studies are represented in Table 1. The classes of antibiotics include Penicillins, sulfonamides, trimethoprim, Pivmecillinam, sulphamethizole macrolides, lincosamides, streptogramins, and other antifungal/antiviral/antibacterial drugs. All included studies had quality levels ranging from moderate [NOS score 4–6] to high [NOS score 7–8] (Table 2). The included studies received quality scores ranging from 6 to 8. In addition, the included studies showed a high risk of comparability bias due to poor confounder adjustment. All included studies are of moderate to high quality, indicating the validity of our findings.

**Table 2:** Quality assessment of the included studies in the systematic review using the Newcastle Ottawa Scale.

**S. No**.	**Author, year**	**Selection**				**Comparability**		**Exposure**			**Quality score**	**Quality grade**
		**Definition of non-exposed group**	**Representativeness of exposed group**	**Selection of Non-exposed**	**Definition of Non-exposed**	**Outcome of interest was not present at the start of study**	**Comparability between the groups**	**Ascertainment of exposure**	**Same method of ascertainment for exposed and non-exposed group**	**Adequacy of follow-up**		
1.	Lavebratt et al. (27)	1	1	1	1	1	0	1	0	1	7	High
2.	Hamad et al. (17)	1	1	1	1	1	1	1	0	1	8	High
3.	Fan et al. (16)	1	1	1	1	1	0	1	0	1	7	High
4.	Leviton et al. (28)	1	1	1	1	1	0	0	0	1	6	Medium
5.	Atladottir et al. (29)	1	1	1	1	1	0	1	0	1	7	High
6.	Guisso et al. (11)	1	1	1	1	1	0	1	0	1	7	High
7.	Isaksson et al. (19)	1	1	1	1	1	0	1	0	1	7	High
8.	Abelson et al. (19)	1	1	1	1	1	0	0	0	1	6	Medium
9.	Hamad et al. (18)	1	1	1	1	1	1	1	0	1	8	High
10.	Sassonker-Joseph et al. (30)	1	1	1	1	1	0	1	0	1	7	High
11.	Miller et al. (31)	1	1	1	1	1	0	1	0	1	7	High
12.	Miller et al. (32)	1	1	1	1	1	0	1	0	1	7	High
13.	O'Shea et al. (34)	1	1	1	1	1	0	0	0	1	6	Medium
14.	Miller et al. (32)	1	1	1	1	1	0	1	0	1	7	High
15.	Meeraus et al. (15)	1	1	1	1	1	0	0	0	1	6	Medium

#### Association between childhood ASD and prenatal antibiotic exposure

**Figure 2:**
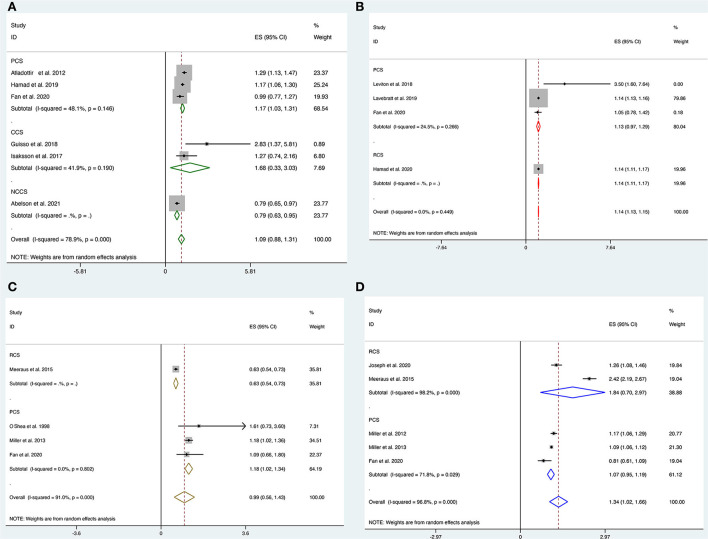
**(A–D)** Forest plot for the association of prenatal exposure to antibiotics and risk of neurodevelopmental disorders in the offspring. **(A)** Risk of ASD. **(B)** Risk of ADHD. **(C)** Risk of CP. **(D)** Risk of epilepsy.

Six studies, including three prospective cohort studies (16, 18, 29), one nested case-control study (20), and two case-control studies (11, 19), explored the link between prenatal maternal antibiotic therapy and the risk of ASD in offspring. Our results showed no overall association between prenatal antibiotic exposure and the risk of ASD [OR = 1.09; 95% CI = 0.88 to 1.31, *I*^2^ = 78.9%]. Only prospective cohort studies found a significant relationship with the pooled OR of 1.17 (95% CI, 1.03–1.31; Figure 2A), with modest evidence of heterogeneity (*P* = 0.14, *I*^2^ = 48.1%) in the subgroup analysis based on the study design.

#### Association between prenatal antibiotic exposure and childhood ADHD

Pooled analysis of four studies involving three prospective (16, 27, 28) and one retrospective (17) cohort studies showed a strong correlation between prenatal antibiotic exposure and the risk of ADHD [OR = 1.14; 95% CI = 1.13 to 1.15, *I*^2^ = 0%]. However, subgroup analysis showed a borderline risk of ASD in the prospective cohort [OR = 1.13; 95% CI = 0.97 to 1.29, *I*^2^ = 24.5%] and a strong association for the risk of ASD in the retrospective cohort [OR = 1.14; 95% CI = 1.11 to 1.17, Figure 2B].

#### Association between prenatal antibiotic exposure and childhood CP

Four studies (15, 16, 33, 34) looked at the link between prenatal antibiotic therapy and the risk of CP in offspring. There was no significant association between prenatal antibiotic exposure and the risk of CP [OR = 0.99, 95% CI = 0.56 to 1.43, *I*^2^ = 91%]. Subgroup analysis suggested a significant association between prenatal antibiotic exposure and the risk of CP [OR = 1.18, 95% CI = 1.02 to 1.34, *I*^2^ = 0%] in all three included prospective cohort studies but not in the retrospective studies [OR = 0.63, 95% CI = 0.54 to 0.73, Figure 2C].

#### Association between prenatal antibiotic exposure and childhood epilepsy during pregnancy

Five studies including three prospective cohort studies (16, 31, 32) and two retrospective cohort studies (15, 30) looked at the link between antibiotic therapy in pregnant women and the risk of epilepsy in offspring. Overall, prenatal antibiotic exposure was significantly associated with the risk of epilepsy [OR = 1.34, 95% CI = 1.02 to 1.66, *I*^2^ = 96.8%]. However, these associations were not significant in the subgroup analysis in both prospective [OR = 1.07, 95% CI = 0.95 to 1.19, *I*^2^ = 71.8%] and retrospective studies [OR = 1.84, 95% CI = 0.70 to 2.97, Figure 2D].

### Publication bias

**Figure 3:**
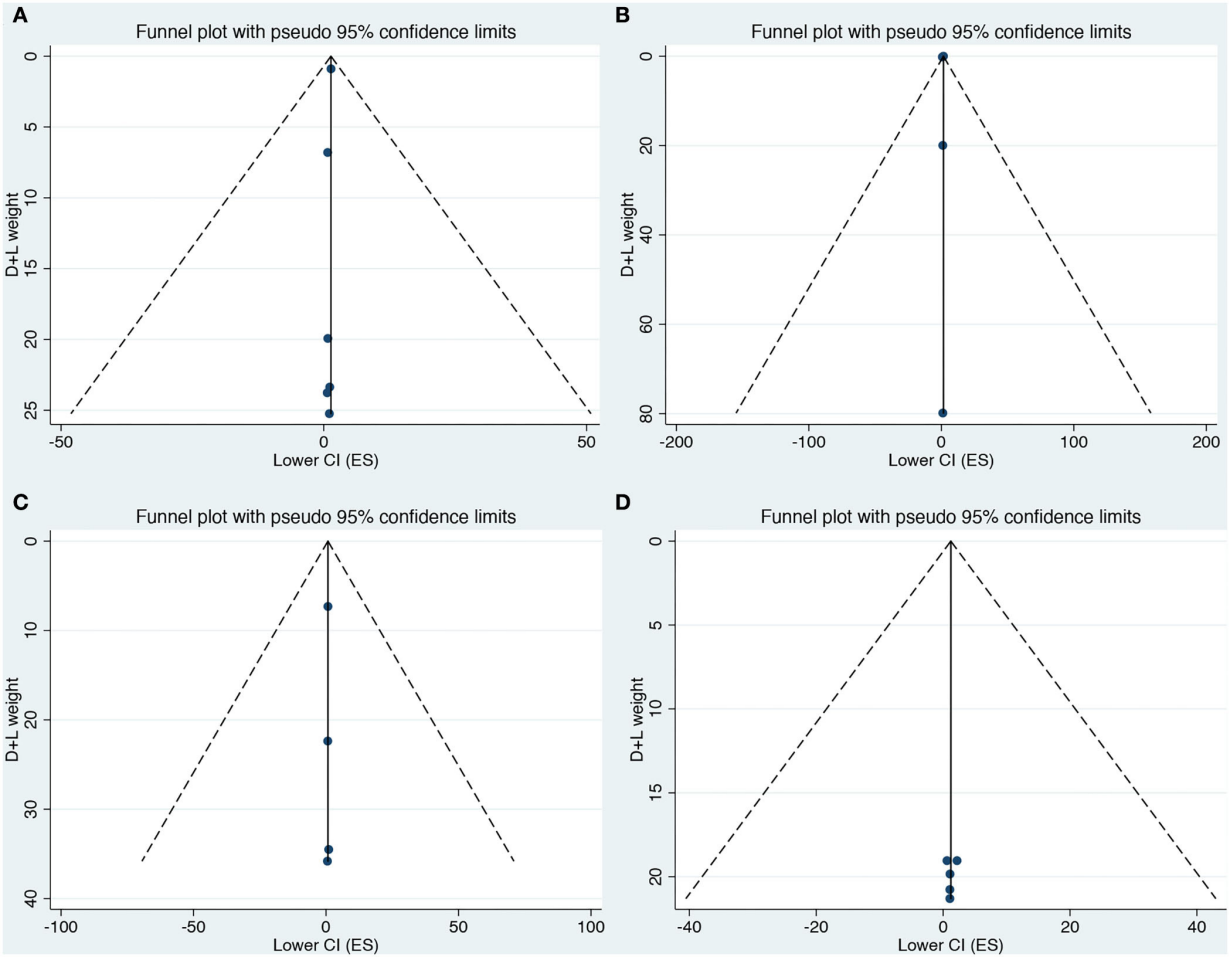
**(A–D)** Funnel plot for the association of prenatal exposure to antibiotics and risk of neurodevelopmental disorders in the offspring. **(A)** Risk of ASD. **(B)** Risk of ADHD. **(C)** Risk of CP. **(D)** Risk of epilepsy.

Egger test and Begg's funnel plot were used to analyzing publication bias for all 15 included studies in our meta-analysis. There was no discernible asymmetry in any included neurodevelopmental disorders based on the visual assessment of the funnel plot (Figures 3A–D). We found no evidence of publication bias in the included studies by Egger's test.

### Sensitivity analyses

**Figure 4:**
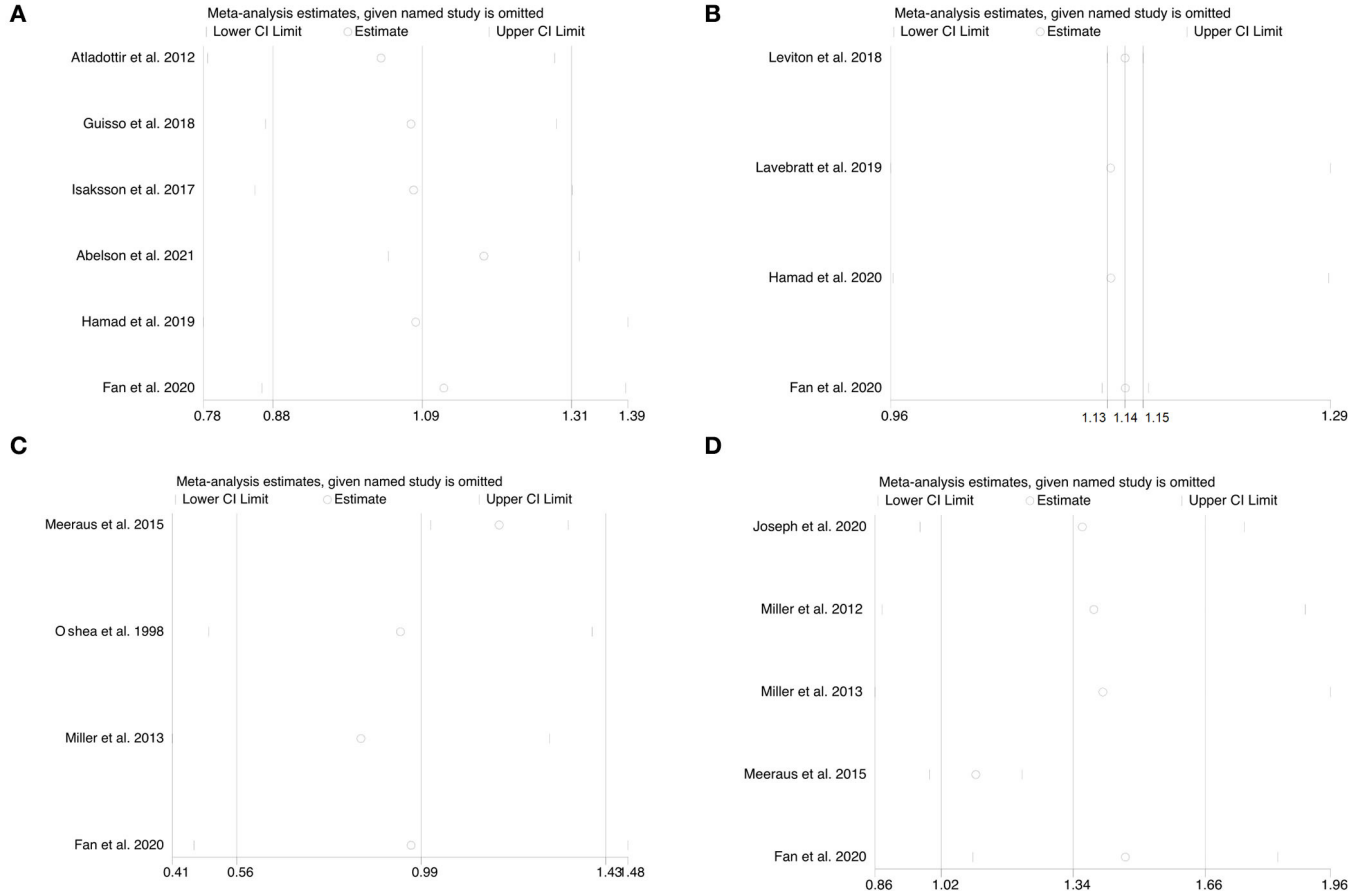
**(A–D)** Sensitivity analysis for the association of prenatal exposure to antibiotics and risk of neurodevelopmental disorders in the offspring. **(A)** Risk of ASD. **(B)** Risk of ADHD. **(C)** Risk of CP. **(D)** Risk of epilepsy.

Sensitivity analyses were performed to see how each individual study of each neurodevelopmental disorder affected the pooled ORs by omitting individual included studies one at a time. Deleting any individual study did not significantly change the associated pooled ORs (Figures 4A–D), further verifying that the findings of the current meta-analysis are statistically sound and robust.

## Discussion

Infections have long been linked to aberrant prenatal brain development, and negative outcomes include cerebral palsy (35), developmental delay (36, 37), epilepsy (38), and schizophrenia (39). However, the reasons behind these associations are still unclear. Several studies suggested that certain maternal infections induce fetal brain impairment (38, 40). Since mothers with these infections are more likely to be treated with antibiotics, maternal infection was considered a confounder in the analyses, and accounting for infections can lead to erroneous results that reflect the infection rather than the therapy. Several meta-analyses have published data on infections that occurred prior to ASD diagnosis (41) and identified a significant positive association of ASD with infections that occurred during pregnancy (42, 43). While one systematic analysis found an elevated risk of ASD after prenatal exposure to various drugs, including antibiotics, the association of the diagnosis with individual drugs was not examined (43). Another meta-analysis revealed no evidence of a link between early postnatal infections and the likelihood of ASD later in life (44). On the other hand, several observational studies have discovered incidences of increased risk of mental illnesses in older children and adults following certain diseases (2, 45). The immune system can be influenced by infections and a dysfunctional gut microbiome. Numerous studies show the role of the immune system, inflammation, and autoimmunity in the etiology of neurodevelopmental and psychiatric disorders in children. Studies showed that the levels of pro-inflammatory cytokines are elevated in children with bipolar and major depressive disorders, posttraumatic stress disorder, obsessive-compulsive disorder, schizophrenia, ADHD, and Tourette's syndrome (46). Immune activation has also been linked to the pathogenesis of major depressive disorder (47) and schizophrenia (48) in adults. Recent studies suggest that immunomodulation plays a key role in orchestrating microbiota–gut–brain communication and has the potential to influence neurodevelopment (49–52).

To the best of our knowledge, this is the first systematic review and meta-analysis to examine the link between antibiotic exposure and the risk of neurodevelopmental disorders such as autism, ADHD, CP, and epilepsy. Our findings show a strong connection between prenatal antibiotic exposure and the risk of ADHD and epilepsy. However, we found no significant association between antibiotic exposure during pregnancy and the risk of ASD and CP. While further subgroup analysis based on the type of studies showed a significant correlation between prenatal antibiotic exposure and the risk of ASD and CP in the included prospective cohorts, no such association was found in retrospective studies.

Our findings are consistent with the previously published meta-analysis by Lee et al. (21) for ASD and by Ai et al. (22) for ADHD. There is no meta-analysis available for the possible association between prenatal antibiotic exposure and the risk of CP and epilepsy.

Lee et al. (21) concluded that prenatal antibiotic exposure may increase the risk of ASD in children. This study confirmed that while the use of antibiotics before and after birth raised the risk of ASD, only prenatal antibiotic use was substantially higher in children with ASD compared to children who were not exposed to prenatal treatment. However, the existing studies on the relationship between ASD risk and postnatal antibiotic use do not specifically focus on prenatal antibiotic exposure, making the comparison or evaluation of the risk levels of pre- and post-natal antibiotic use difficult. Another meta-analysis by Ai et al. (22) which included 11 studies suggested the association of prenatal antibiotic treatment with an increased risk of ADHD in children. However, there was a high degree of heterogeneity and publication bias in their analysis which may be due to the large disparity in population characteristics, sample size, timing of antibiotic administration, and antibiotic kind. The precision of the reported pooled estimations was also low which limits the applicability of their findings.

In the present meta-analysis, we found a substantial association between prenatal antibiotic exposure and the risk of CP and ASD, as well as an overall association with ADHD and epilepsy. Our results show a strong association between prenatal antibiotic exposure with the increased risk of ADHD and epilepsy development. While our combined results did not indicate that there is a substantial relationship between maternal antibiotic treatment and incidences of ASD and CP in offspring, this association was significant in prospective cohort studies. This could be because neurodevelopmental diseases have multiple causes. For example, non-genetic causes account for 70% of cerebral palsy cases, whereas genetic variables account for 60–70% of epilepsy, ADHD, and ASD cases (53–56).

Our systematic review and meta-analysis have some limitations, and therefore, results must be interpreted with caution. Only a limited number of published studies investigated a high degree of heterogeneity between study outcomes. This may be due to the inclusion of studies with different study designs leading to recall bias for the observed findings. Variability in the available outcomes, such as the type, number, and timing of antibiotic exposures among the included studies, limited our ability to perform further subgroup analyses. Due to the lack of genetic predisposition data, we were not able to correlate our findings with antibiotic exposure.

## Conclusion

Our data show that prenatal antibiotic exposure during pregnancy is linked to a higher incidence of ADHD and epilepsy in children. Further prospective studies, comparing prenatal antibiotic use with adjustment for various confounders, are needed.

## Data availability statement

The original contributions presented in the study are included in the article/supplementary material, further inquiries can be directed to the corresponding authors.

## Author contributions

QT and YS analyzed the data and contributed to the writing of the manuscript. YL, HL, and MY contributed to the data collection and analysis. JG contributed to the substantial review and editing of the manuscript. All authors read and approved the final manuscript.

## Funding

This study was funded by a grant from the National Natural Science Foundation of China (No. 82071686), the Science and Technology Bureau of Sichuan province (No. 2021YFS0093), and the Research Fund of West China Second University Hospital (No. KL115, KL072).

## Conflict of interest

The authors declare that the research was conducted in the absence of any commercial or financial relationships that could be construed as a potential conflict of interest.

## Publisher's note

All claims expressed in this article are solely those of the authors and do not necessarily represent those of their affiliated organizations, or those of the publisher, the editors and the reviewers. Any product that may be evaluated in this article, or claim that may be made by its manufacturer, is not guaranteed or endorsed by the publisher.
